# From Fragmentation to Standardization: A Systematic Review Update and Critical Appraisal of Genetic and Genomic Testing Assessment Frameworks and Their Alignment With the HTA Core Model

**DOI:** 10.1002/hsr2.73446

**Published:** 2026-10-28

**Authors:** Valentina Baccolini, Antonio Sciurti, Erica Pitini, Leonardo Maria Siena, Arianna Anniballo, Anna Ewa Kaminska, Andrea Pistollato, Romina Pausilli, Chiara Tommasino, Carolina Marzuillo, Corrado De Vito, Giuseppe La Torre, Giuseppe Migliara, Paolo Villari

**Affiliations:** ^1^ Department of Public Health and Infectious Diseases Sapienza University of Rome Rome Italy; ^2^ Department of Translational and Precision Medicine Sapienza University of Rome Rome Italy; ^3^ Department of Life Sciences, Health, and Health Professions Link Campus University Rome Italy; ^4^ Independent Researcher Rome Italy; ^5^ UniCamillus ‐ Saint Camillus International University of Health Sciences Rome Italy

**Keywords:** biomedical, genetic testing, precision medicine, technology assessment

## Abstract

**Background and Aims:**

Owing to their distinct features, genetic/genomic technologies have traditionally been assessed using tailored approaches, with no standardized methodology emerging to date. The Health Technology Assessment (HTA) Core Model (version 3.0), adopted within the European Union HTA Regulation, is a rigorous framework for health technology assessment, but its suitability for genetic/genomic testing remains unclear. This study examined whether the Model could serve as a standardized framework for the evaluation of genetic/genomic applications.

**Methods:**

A search was conducted to identify evaluation frameworks for genetic/genomic applications (PROSPERO CRD42023429403), and a critical analysis was performed. The methodological characteristics of the identified frameworks, including their assessment domains and components, were systematically compared to the domains and issues of the HTA Core Model. Findings were synthesized narratively.

**Results:**

Overall, 24 evaluation frameworks for genetic/genomic testing were analysed: half were based on the ACCE model, four on the HTA approach, one combined both methodologies, and seven were heterogeneous in structure. Only one framework covered all relevant assessment aspects but lacked validation. Conversely, the HTA Core Model captured all core questions and addressed every identified assessment component, although with varying degrees of coverage (Cohen's κ between researchers: 0.71, 95% CI: 0.59–0.82).

**Conclusion:**

Existing frameworks for genetic/genomic technologies are fragmented and mostly incomplete. As the HTA Core Model encompasses all relevant components of these technologies, it may offer a suitable basis for standardizing their evaluation process. Future research could integrate it with a few genomic‐specific items to maximize its exhaustiveness.

## Key Points

- This study addresses the lack of standardized approaches for assessing genetic and genomic technologies by systematically reviewing existing evaluation frameworks and comparing their characteristics with the HTA Core Model (version 3.0).
- Results indicate that current frameworks are fragmented and incomplete, but every relevant assessment component is addressed within the HTA Core Model, confirming its applicability to these technologies.
- Adopting the HTA Core Model as a common framework can strengthen healthcare decision‐making for genetic and genomic technologies by enabling standardized and transparent evaluation of these applications.

## 1 Introduction

As genetic and genomic applications become increasingly accessible, there is a growing demand for their use in clinical and public health practice [1], and rigorous and transparent assessment is essential for responsible and effective implementation of such innovative technologies, particularly in publicly funded healthcare systems, where finite budgets require priority setting among healthcare and non‐healthcare demands [2]. The ACCE model was one of the first evidence‐based frameworks developed specifically to assess genetic testing [3]. It takes its name from the four evaluation dimensions considered (i.e., Analytic validity, Clinical validity, Clinical utility, and Ethical, legal, and social implications) and operationalize them through a structured set of 44 guiding questions to synthesize evidence and flag gaps in knowledge [3], features that were designed to support both rapid and comprehensive appraisals of genetic tests [4].

From 2010 onwards, numerous other theoretical frameworks have been developed to support the evaluation of these technologies, some grounded in the ACCE model and a few adopting a more generic Health Technology Assessment (HTA) approach [5], but no single standardized methodology has emerged [6]. This fragmentation has resulted in non‐comparable assessments of these technologies that, coupled with varying degrees of depth and coverage of key evaluation domains, may have complicated their thoughtful integration into clinical practice [7, 8]. In the same period, the European Network for Health Technology Assessment (EUnetHTA) designed the HTA Core Model, one of the most rigorous frameworks for evaluating health technologies in general [9, 10]. Its first version was developed between 2006 and 2008 and subsequently updated in 2013 (version 2.0) and 2016 (version 3.0), through EUnetHTA Joint Actions (JA) 1 and 2, respectively [11]. JA2 also aimed to facilitate the application of the Model to cross‐border HTA collaboration, while JA3 (2016–2021) focused on defining and further validating the collaborative approach. The outcomes of JA3 ultimately provided evidence in favor of the need for EU‐level support, contributing to the European Commission's proposal for an HTA Regulation in 2018 and its subsequent adoption in 2021 [11, 12]. The HTA Core Model therefore provided an important methodological foundation for the Regulation's Joint Clinical Assessments (JCAs), which establish a common EU‐level assessment of the clinical domains of health technologies conducted by the Member State Coordination Group on HTA (HTACG), while leaving other aspects of HTA to individual Member States HTA agencies. In this way, the JCA framework aims to reduce duplication and support more consistent, evidence‐based decision‐making across EU countries [11].

Indeed, a standardized evaluation framework for genetic and genomic technologies is likely to reduce the proliferation of heterogeneous and non‐comparable assessments, as previously observed [6]. Standardized assessments conducted by different institutions would enable HTA agencies to build on each other's work, thereby reducing duplication of effort, one of the main objectives of European HTA cooperation [12]. Furthermore, a shared framework could support policymakers in promoting the uptake of high‐value tests while limiting the implementation of applications with uncertain value–particularly in genetics and genomics, where conditions are often rare and the evidence base is limited and fragmented [7]. This could contribute to more consistent clinical and public health decision‐making, improve equity in access to effective genomic technologies, and facilitate more efficient allocation of healthcare resources [13].

Building on these premises, the HTA Core Model has been proposed as a potential foundation for a standardized framework for evaluating genetic and genomic technologies, while retaining a general HTA approach. However, to the best of our knowledge, this potential has not yet been systematically assessed [8, 14]. Therefore, the objective of this study was to appraise whether the HTA Core Model could serve as a framework for evaluating genetic and genomic technologies, by comparing its methodological characteristics with those of existing evaluation frameworks specifically designed for genetic and genomic applications.

## 2 Methods

To establish whether the HTA Core Model could be suitable as a general framework for the assessment of genetic and genomic application, we (i) critically reviewed and analysed existing evaluation frameworks for genetic and genomic applications and (ii) systematically compared their key methodological characteristics with those of the HTA Core Model.

## 3 Critical Review and Analysis of Existing Evaluation Frameworks for Genetic and Genomic Applications

To collect all existing evaluation frameworks for genetic and genomic applications, given that two systematic reviews had already been conducted in 2018 and 2021 [7, 8], we updated the most recent review [8] in accordance with the Cochrane Handbook for systematic reviews [15] and we adhered to the Preferred Reporting Items for Systematic Reviews and Meta‐Analyses (PRISMA) statement for its reporting [16]. The review protocol was registered at PROSPERO (identifier: CRD42023429403). Since this study did not involve primary data collection, informed consent from participants was not required and the protocol was not submitted for institutional review board approval.

### 3.1 Search Strategy, Study Selection and Inclusion Criteria

We replicated the same search and screening strategies used in the previous reviews [7, 8]. Accordingly, PubMed, Scopus, Web of Science and Google Scholar were searched from October 1, 2020, the completion date of the previous systematic searches [8], to May 7, 2025, using key terms related to genetic/genomic tests and evaluation frameworks (Supporting Information S1: Table 1). Duplicate records were eliminated, and two reviewers screened all records to identify those that might meet the inclusion criteria. Documents that were identified by previous reviews but were unretrievable at the time of the current update were dismissed and not considered for inclusion.

This review included any document reporting (i) any assessment framework for genetic/genomic technologies on human specimens and for human conditions [17], that was (ii) original, i.e., was expressly created for this purpose; (iii) was deemed complete, meaning that it comprised at least three assessment aspects; and (iv) was reported in English or Italian, based on coauthor language abilities. We excluded documents that were written in languages other than English or Italian; that were editorials, reviews, book chapters, conference abstracts; and that were not available as a full text.

### 3.2 Data Collection and Synthesis

As in the previous systematic reviews, two reviewers independently extracted the following information for each assessment framework: identification (i.e., authors, year of publication, country of publication, and institution/organization responsible for producing the framework); general framework information (i.e., framework name, reference frameworks, target genetic/genomic tests [i.e., single‐gene, multiple‐gene, or genomic tests] [18], purpose, and primary audience). To allow comparability with the HTA Core Model, additional information on the frameworks’ methodological characteristics was identified that had not been extracted in previous systematic reviews, including assessment domains and their components, as well as reporting aspects (i.e., quality of the evidence assessment and reporting of evidence gaps/research priorities).

For the purposes of this study, we refer to assessment domain as broader areas of focus that encompass multiple assessment components, while assessment components are the specific pieces of information that frameworks recommend considering when evaluating genetic and genomic technologies within each assessment domain. The following assessment domains and their components were identified: (i) “disease/condition of interest”, components: epidemiology, etiopathogenesis, natural history, current management; (ii) ‘genetic/genomic application’, components: classification (i.e., predictive, presymptomatic, carrier, diagnostic, pharmacogenetic, prognostic tests) [18], type of technology, analytical accuracy; (iii) ‘clinical aspects’, components: test purpose (i.e., prediction, screening, diagnosis, treatment, prognosis), target population, clinical setting, post‐test intervention, post‐test clinical pathways, efficacy, effectiveness, safety, patient‐reported outcomes, clinical accuracy; (iv) “personal and societal aspects,” components: non‐health‐related outcomes, ethical implications, legal implications, social implications; (v) “organizational aspects,” components: burden of disease, professional resources needs, material resources needs, financial resources needs, economic assessment, equity in access, barriers to implementation.

Information extracted was descriptively summarized in tables and figures, and frameworks were grouped into four categories based on their structure: (i) frameworks based on the ACCE framework (“ACCE only”); (ii) frameworks based on a HTA framework (“HTA only”); (iii) frameworks based on a combination of both ACCE and HTA frameworks (“ACCE + HTA”); and (iv) other frameworks.

### 3.3 Comparison Between the Assessment Domains/Components of the Identified Evaluation Frameworks and the Assessment Domains/Issues of the HTA Core Model

The HTA Core Model (version 3.0) [19] organizes the information necessary for technology assessment into a hierarchical structure. At the highest level, there are nine different domains: (i) health problem and current use of technology; (ii) description and technical characteristics of technology; (iii) safety; (iv) clinical effectiveness; (v) costs and economic evaluation; (vi) ethical analysis; (vii) organizational aspects; (vii) patients and social aspects; (ix) legal aspects. Each domain is then divided into topics, and each topic is further divided into issues. These issues are the smallest assessment pieces, representing the questions that should be considered when assessing health technology, and there are 136 unique issues in total. In this study, two authors independently linked the assessment domains and components identified across the frameworks in the systematic review to the specific assessment domains and issues from the HTA Core Model (version 3.0). Cohen's κ statistic and its corresponding 95% Confidence Interval (CI) was used to measure the agreement between the authors' judgements [20] and conflicts were resolved by consensus.

All analyses were performed with R Statistical Software (version 4.2.3; R Core Team 2023, R Foundation for Statistical Computing, Vienna, Austria).

## 4 Results

### 4.1 Critical Review and Analysis of Existing Evaluation Frameworks for Genetic and Genomic Applications

A total of 23,611 new unique records were identified through the systematic review update [8] and 66 full‐text documents were assessed for eligibility. However, none of these met the inclusion criteria. Moreover, eight documents reporting six original assessments frameworks from the previous systematic reviews were dismissed as non‐retrievable. As a result, 29 documents reporting 24 original assessment frameworks [3, 4, 5, 21, 22, 23, 24, 25, 26, 27, 28, 29, 30, 31, 32, 33, 34, 35, 36, 37, 38, 39, 40, 41, 42, 43, 44, 45, 46, 47] were considered eligible for our analysis and included in our review (Figure 1).

**Figure 1:**
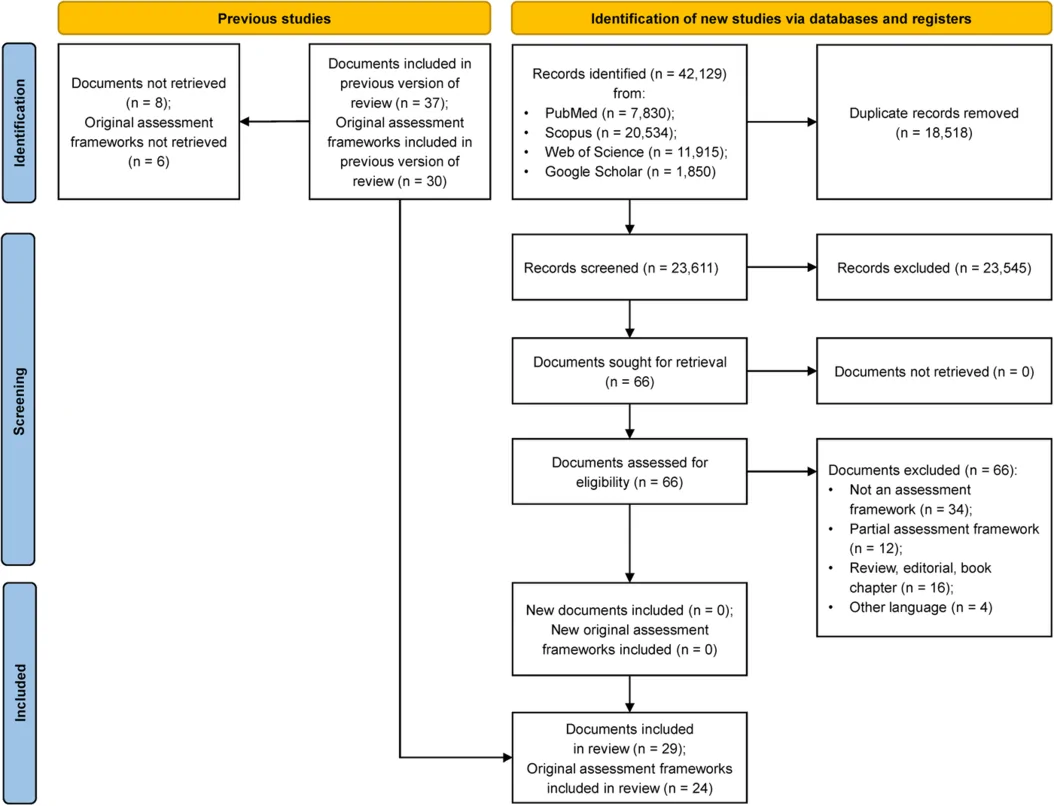
Flow diagram for systematic review update process.

### 4.2 General Characteristics of the Evaluation Frameworks

General characteristics of the assessment frameworks are shown in Table 1. Frameworks span multiple countries, with the majority originating from the United States (12 frameworks, 50.0%) [3, 4, 21, 22, 23, 24, 25, 26, 29, 31, 32, 33, 39, 42, 43, 44], followed by Canada and the United Kingdom (four, 33.3% [21, 22, 27, 28, 37], and three, 12.5% [5, 45, 46, 47], respectively), Australia and Spain (two each) [30, 34, 36, 38]. The frameworks were developed from 2001 to 2019, with most published between 2010 and 2014 (12 frameworks, 50.0%) [21, 22, 23, 24, 26, 30, 32, 33, 36, 41, 42, 43, 44, 47].

**Table 1:** General characteristics of genetic/genomic testing assessment frameworks (*N* = 24).

Author, year	Country	Institution	Framework name	Reference framework	Target test	Purpose	Primary audience
Andermann et al. 2010 [21] Andermann et al. 2011 [22]	Canada; USA	McGill University; Princeton University	Genetic Screening	Wilson and Jungner criteria	Genetic tests in screening programs	PM	Policy makers
Burke & Zimmern 2007 [5]	UK	PHGF	Expanded ACCE	ACCE	Genetic tests	CG, PM	Policy makers, health professionals, patients
Burke et al. 2001 [23]	USA	Washington University	Population screening for adult‐onset conditions	Wilson and Jungner criteria	Genetic tests in screening programs	PM	Policy makers
Calonge et al. 2010 [24]	USA	ACHDNC	ACHDNC	ACCE	Genetic tests in screening programs	PCD	Policy makers
Canestaro et al. 2015 [25]	USA	Washington University	CAT	ACCE	Pharmacogenetic tests	CG	Policy makers, health professionals
CDC 2002 [31], Haddow & Palomaki 2004 [3]	USA	CDC	ACCE	ACCE	Genetic tests	CG, PM	Policy makers, health professionals, general public
Frueh & Quinn 2014 [26]	USA	Opus Three	Six‐part framework	ACCE	Genetic tests	PCD	Policy makers
Giacomini et al. 2003 [27]	Canada	McMaster University	McMaster Model	HTA	Predictive genetic tests	PCD	Policy makers
Goel 2001 [28]	Canada	Crossroads 99 group	Crossroads 99	Wilson and Jungner criteria	Genetic tests in screening programs	PM	Policy makers
Gudgeon et al 2007 [4], McClain et al. 2008 [29]	USA	Brown University	Rapid ACCE	ACCE	Genetic tests	CG, PM	Policy makers, health professionals, patients
Gutiérrez‐Ibarluzea 2012 [30]	Spain	OSTEBA	HTA Personalized Health Care	HTA	Genomic tests	PCD	Policy makers
Hornberger et al. 2012 [32]	USA	Stanford University; Cedar Associates	SynFRAME	Various	Genetic tests	CG	Policy makers, health professionals, patients
Lin et al. 2012 [33]	USA	KPNW	Practical framework	Not reported	Genetic tests	CG, PM	Policy makers, health professionals, patients
MSAC 2016 [34]	Australia	MSAC	Clinical Utility Card	ACCE	Genetic tests	PCD	Policy makers
Merlin et al. 2013 [36]	Australia	AHTA	Framework for co‐dependent technologies	HTA	Genetic tests	PCD	Policy makers
Mykitiuk 2001 [37]	Canada	PACNPGT	PACNPGT	HTA	Pharmacogenetic tests	PCD	Policy makers
Márquez Calderón & Briones Pérez de la Blanca 2006 [38]	Spain	AETSA	Andalusian	ACCE	Predictive genetic tests	PCD	Policy makers, health professionals
NASEM 2017 [39]	USA	NASEM	Evidence framework for genetic testing	Various	Genetic tests	PM	Policy makers
Pitini et al. 2019 [40]	Italy	Sapienza University	Sapienza	ACCE + HTA	Genetic tests	PM	Policy makers
Rousseau et al. 2010 [41]	International	IFCC	GETT	ACCE	Genetic tests	PCD	Policy makers, health professionals
Sun et al. 2011[42]	USA	ECRI	ECRI	Various	Genetic tests	PCD	Policy makers, health professionals, patients
Teutsch et al. 2009 [43], Veenstra et al 2013 [44]	USA	EGAPP	EGAPP	ACCE	Genetic/genomic tests	CG, PM	Policy makers, health professionals
UKGTN 2005 [45], Kroese et al. 2007 [46]	UK	UKGTN	UKGTN Gene Dossier	ACCE	Genetic tests	PCD	Policy makers
Wright & Kroese 2010 [47]	UK	PHGF	Complex Diseases	ACCE	Predictive genetic tests	CG, PM	Policy makers, health professionals, general public

Abbreviations: ACCE, analytic validity, clinical validity, clinical utility, ethical, legal and social implications; ACHDNC, Advisory Committee on Heritable Disorders in Newborns and Children; AETSA, Andalusian Health Technology Assessment Department; AHTA, Adelaide Health Technology Assessment; CAT, Companion Tests Assessment Tool; CDC, Centers for Disease Control and prevention; CG, Clinical Guidance; ECRI, Emergency Care Research Institute; EGAPP, Evaluation of Genomic Applications in Practice and Prevention; GETT, Genetic testing Evidence Tracking Tool; HTA, Health Technology Assessment; IFCC, International Federation of Clinical Chemistry and Laboratory Medicine; KPNW, Kaiser Permanente of the Northwest; MSAC, Medical Service Advisory Committee; NASEM, National Academies of Sciences; OSTEBA, Basque Office for Health Technology Assessment; PACNPGT, Provincial Advisory Committee on New Predictive Genetic Technologies; PHGF, Public Health Genomics Foundation; PCD, Provision or Coverage Decision; PM, Policy Making; UKGTN, UK Genetic Testing Network.

Regarding the reference framework, ACCE framework was the most predominant (12 frameworks, 50.0%) [3, 4, 5, 24, 25, 26, 29, 31, 34, 38, 41, 43, 44, 45, 46, 47], followed by the HTA framework (4 frameworks, 33.3%) [27, 30, 36, 37] and the Wilson and Jungner criteria (3 frameworks, 12.5%) [21, 22, 23, 28]. Only one framework combined the ACCE and HTA framework as a reference (Sapienza) [40].

The target tests assessed by these frameworks include mostly genetic tests in general (13 frameworks, 54.2%) [3, 4, 5, 26, 29, 31, 32, 33, 34, 38, 39, 40, 41, 42, 45, 46], followed by genetic tests in screening programs (4 frameworks, 16.7%) [21, 22, 23, 24, 28], predictive genetic tests (3 frameworks, 12.5%) [27, 37, 47], and pharmacogenetic tests (2 frameworks, 8.3%) [25, 36]. Only two frameworks (8.3%) expressly considered genomic tests [30, 43, 44].

Framework purposes were mostly provision and coverage decisions (11 frameworks, 45.8% [24, 26, 27, 30, 34, 36, 37, 38, 39, 41, 42, 45, 46] or generic policy making (5 frameworks, 20.8%) [21, 22, 23, 28, 39, 40], although some frameworks also aimed to inform clinical guidance (8 frameworks, 33.3%) [3, 4, 5, 25, 29, 31, 32, 33, 43, 44, 47]. The primary audiences for these frameworks varied but were always comprised of policymakers [3, 4, 5, 21, 22, 23, 24, 25, 26, 27, 28, 29, 30, 31, 32, 33, 34, 35, 36, 37, 38, 39, 40, 41, 42, 43, 44, 45, 46, 47], with most also addressing health professionals (11 frameworks, 45.5%) [3, 4, 5, 25, 29, 31, 32, 33, 38, 41, 42, 43, 44, 47], and some including patients (5 frameworks 20.8%) [4, 5, 29, 32, 33, 42] and also the general public (2 frameworks, 8.3%) [3, 31, 47].

### 4.3 Assessment Domains and Components of the Evaluation Frameworks

Assessment domains and components of the retrieved frameworks by the reference framework are presented in Figure 2 (see Supporting Table S1: Table S2 for further detail). The only one framework combining ACCE and HTA models (Sapienza) [40] considered all identified components across the assessment domains. Regarding the other frameworks, as for disease/condition of interest domain, the frameworks based on ‘ACCE only’ and ‘HTA only’ frequently addressed epidemiology (91.7% and 75.0%, respectively), while current management, natural history, and etiopathogenesis were less commonly considered. A similar performance was found for the ‘other frameworks’ group.

**Figure 2:**
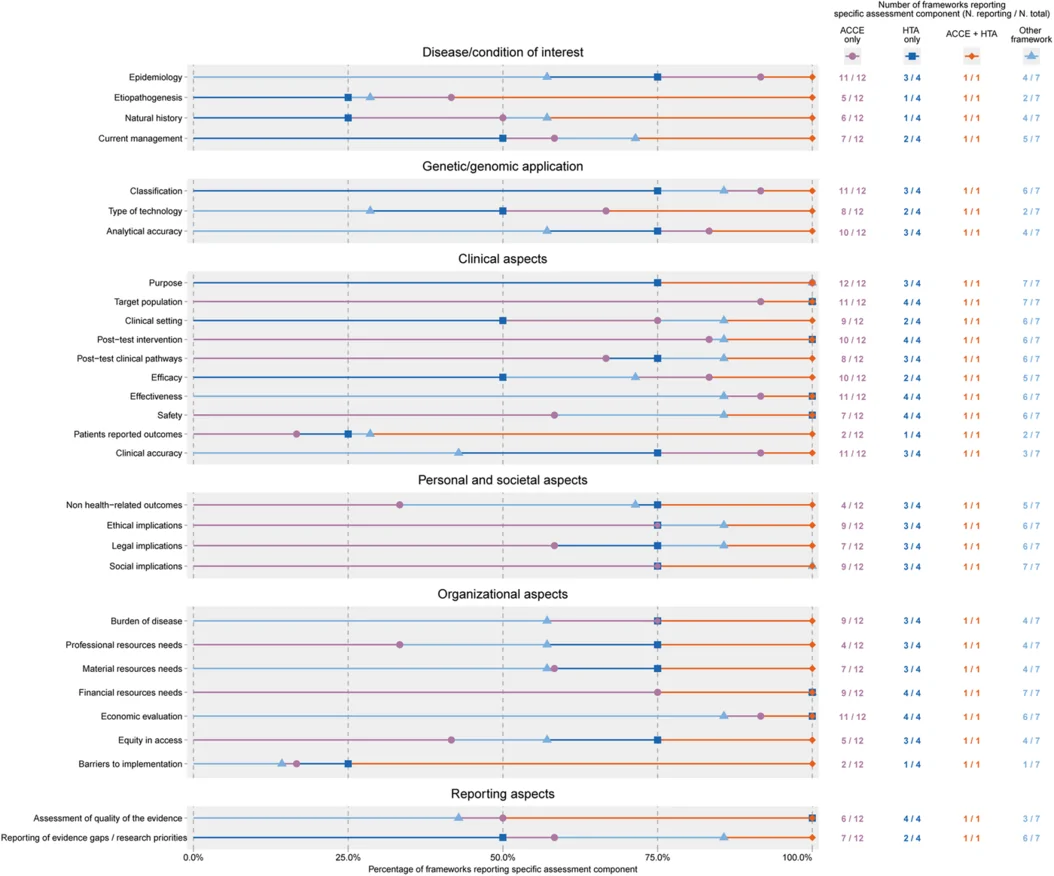
Assessment domains and components by reference framework (“ACCE only,” *N* = 12; “HTA only” *N* = 4; “ACCE + HTA,” *N* = 1; “Other framework,” *N* = 7).

In the genetic/genomic application domain, the classification of the genetic/genomic application was consistently included by the “ACCE only” (91.7%), other reference (85.7%) and the “HTA only” (75.0%) frameworks. Similarly, analytical accuracy was addressed by the majority of ‘ACCE only’ (83.3%) and ‘HTA only’ frameworks (75.0%). In contrast, the type of technology was less consistently covered, being included in 66.7% of “ACCE only” and 50.0% of ‘HTA only’ frameworks.

Within the clinical aspects' domain, ‘ACCE only’ frameworks consistently included test purpose (100%), with most also addressing target population, effectiveness, and clinical accuracy (91.7%). Components such as clinical setting, post‐test intervention, clinical pathways, efficacy, and safety were included by 58.3% to 83.3% of these frameworks. All ‘HTA only’ frameworks (100%) addressed target population and post‐test intervention, effectiveness, safety, while test purpose, post‐test clinical pathways and clinical accuracy were addressed by 75.0% “HTA only” frameworks. Overall, patient‐reported outcomes were the least considered component, ranging from 16.7% in ‘ACCE only’ frameworks to 28.6% in other reference frameworks.

Regarding personal and societal aspects, “ACCE only” frameworks predominantly included ethical and social implications (75.0% each), whereas legal implications and non‐health‐related outcomes were less frequently addressed (58.3% and 33.3%, respectively). In contrast, “HTA only,” and other frameworks included ethical, legal, and social implications in over 70% of cases.

In the organizational aspects' domain, “ACCE only” frameworks frequently considered burden of disease (75.0%), financial resources needs (75.0%), and economic evaluations (91.7%). Components such as material resources needs, professional resources needs, and equity in access were less frequently covered (58.3%, 33.3%, and 41.7%, respectively). ‘HTA only’ frameworks consistently included financial resources needs and economic evaluations (100%), while burden of disease, professional and material resources need and equity in access were included in 75.0% of cases. Frameworks based on other models always included financial resources (100%) and frequently addressed economic evaluations (85.7%), while burden of disease, professional and material resources needs, and equity in access were less frequently considered (57.1% each). Barriers to implementation was the most overlooked component, reported by only 25.0% of the ‘HTA only’ frameworks, 16.7% of ‘ACCE only’ frameworks, 14.3% of other reference frameworks.

Assessment of the quality of evidence was recommended by all ‘HTA only’ (100%) and Sapienza framework (“ACCE + HTA”), but only by 50.0% of ‘ACCE only’ and 42.9% of other frameworks. Conversely, reporting of evidence gaps or research priorities was addressed by Sapienza framework, 85.7% of other reference frameworks, 58.3% of ‘ACCE only’ and 50.0% of ‘HTA only’ frameworks.

### 4.4 Comparison Between the Identified Assessment Domains/Components of the Evaluation Frameworks and the Assessment Domains/Issues of the HTA Core Model

The alignment between the assessment domains and components identified in the analysis of genetic/genomic test frameworks and the specific assessment issues from the HTA Core Model (version 3.0) is presented in Table 2 and further illustrated in Figure 3. Cohen's κ for agreement between reviewers was 0.71 (95% CI: 0.59–0.82). Within the disease/condition domain identified in the systematic review, 12 issues from HTA Core Model were mapped. Most issues related to the current management of the condition (66.7%), with others touching on epidemiology, etiopathogenesis, and natural history. The genetic/genomic application domain featured five issues from the HTA Core Model (version 3.0), with three issues (60.0%) corresponding to the type of technology component. The clinical aspects domain was the most extensively populated, with 90 assessment issues from the Core Model matched to the components retrieved in the systematic review. Issues were predominantly mapped to effectiveness and safety (both 20%), efficacy (18.9%) and clinical accuracy (11.1%). Within the personal and societal aspects domain, 52 HTA Core Model issues were matched, with legal and ethical implications being the most represented (36.5% and 28.8%, respectively). Finally, organizational aspects were the second most represented domain, with 59 issues from the HTA Core Model (version 3.0). Material resources needs and barriers to implementation were the most commonly linked components (20.3% each), followed by equity in access (18.6%), professional resources needs and economic evaluation (15.3% and 13.6%, respectively). Finally, disagreements between reviewers were limited to 20 issues of the HTA Core Model. Of these, 23.8% fell within the “Clinical Effectiveness” domain and 23.8% within the “Organizational Aspects” domain, followed by the “Health Problem and Current Use of the Technology” domain (19.0%), the “Description and Technical Characteristics of the Technology” domain (14.3%), “Ethical Analysis” (9.5%), and the “Patient and Social Aspects” and “Costs and Economic Evaluation” domains (4.8% each).

**Table 2:** Identified assessment components and domains, linked to specific HTA Core Model assessment issues. The same issue may match multiple identified assessment components.

Identified domain and component	HTA Core Model assessment issue ID	N. issues (%)
**Disease/condition of interest (*N*** = **12)**		
Current management	A0005, A0011, A0012, A0018, A0024, A0025, F0001, G0009	8 (66.7)
Epidemiology	A0003	1 (8.3)
Etiopathogenesis	A0003	1 (8.3)
Natural history	A0002, A0004	2 (16.7)
**Genetic/genomic application (*N*** = **5)**		
Analytical accuracy	B0018	1 (20.0)
Classification	B0001	1 (20.0)
Type of technology	B0001, B0003, A0022	3 (60.0)
**Clinical aspects (*N*** = **90)**		
Clinical accuracy	C0006, D1001, D1002, D1003, D1004, D1005, D1006, D1007, D1008, D1019	10 (11.1)
Clinical setting	A0001, B0004, D1007, H0203	4 (4.4)
Effectiveness	B0002, D0001, D0005, D0006, D0010, D0011, D0014, D0015, D0016, D0020, D0021, D0022, D0026, D0029, D0032, E0005, F0010, H0100	18 (20.0)
Efficacy	B0002, D0001, D0005, D0006, D0010, D0011, D0014, D0015, D0016, D0020, D0021, D0022, D0026, D0029, D0032, E0005, F0010	17 (18.9)
Patients‐reported outcomes	D0012, D0013, D0014, D0015, D0016	5 (5.6)
Post‐test clinical pathways	C0006, D0022, D0026, G0001, H0202, H0203	6 (6.7)
Post‐test intervention	D0024, D0026, G0001, H0203	4 (4.4)
Purpose	A0001, A0020, G0009	3 (3.3)
Safety	C0002, C0004, C0005, C0006, C0007, C0008, C0020, C0040, C0062, C0063, C0064, D0011, D0014, D0015, D0016, D1019, E0005, F0010	18 (20.0)
Target population	A0001, A0007, A0023, G0009, G0100	5 (5.6)
**Personal and societal aspects (*N*** = **52)**		
Ethical implications	F0003, F0004, F0005, F0006, F0007, F0008, F0009, F0013, F0014, F0016, F0017, F0101, F0102, F0103, F0104	15 (28.8)
Legal implications	F0003, F0014, F0016, I0002, I0007, I0008, I0009, I0011, I0012, I0015, I0017, I0019, I0021, I0023, I0024, I0025, I0026, I0034, I0037	19 (36.5)
Non‐health‐related outcomes	D0017, D0030, F0003, F0010, F0011, H0002, H0006, H0200	8 (15.4)
Social implications	A0006, F0003, F0005, F0009, F0012, G0101, H0002, H0006, H0012, H0201	10 (19.2)
**Organizational aspects (*N*** = **59)**		
Burden of disease	A0009	1 (1.7)
Professional resources needs	B0012, B0013, D0023, E0001, E0002, E0009, G0003, G0004, G0005	9 (15.3)
Material resources needs	B0007, B0008, B0009, B0010, B0012, D0023, E0001, E0002, E0009, G0005, G0006, G0012	12 (20.3)
Financial resources needs	D0023, E0001, E0002, E0009, G0005, G0007	6 (10.2)
Economic evaluation	A0021, E0005, E0006, E0010, E0011, E0012, E0013, F0102	8 (13.6)
Equity in access	A0012, B0014, B0015, F0012, G0002, G0011, G0101, H0012, H0201, I0011, I0012	11 (18.6)
Barriers to implementation	B0014, B0015, C0040, C0062, C0063, C0064, F0104, G0002, G0008, G0010, G0011, H0203	12 (20.3)

**Figure 3:**
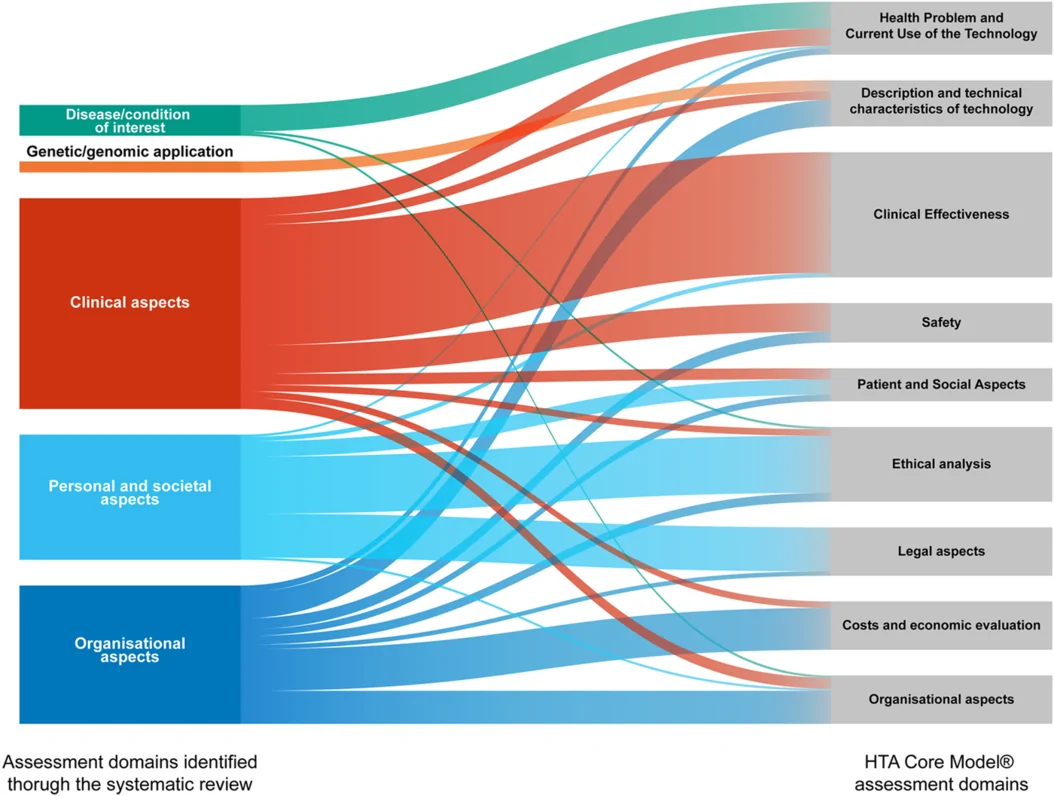
Comparison between the assessment domains identified through the systematic review (nodes on the left) and the EUnetHTA HTA Core Model assessment domains (nodes on the right). The edges' thickness is weighted according to the number of the corresponding Core Model assessment issues.

## 5 Discussion

In the field of genomic medicine, the absence of clear, standardized, and transparent evaluation processes has been identified as a key barrier to implementation, contributing to heterogeneous assessments and slower translation of innovative applications into routine practice [48, 49]. This challenge also extends to other technologies: in a state‐of‐the‐art review of HTA methods guidance for diagnostic tests, Ferrante di Ruffano et al. investigated international HTA methods guidance with the explicit aim of identifying and sharing good practice [50]. Similarly, other authors used an international survey to map current practices in the HTA of omics technologies [51]. In our attempt to identify a standardized evaluation framework for genetic and genomic technologies, we started with an update of previous systematic reviews [7, 8] to critically analyze existing approaches. Notably, in this update, we did not identify any newly published framework specifically designed for the evaluation of genetic and genomic tests. This may be explained by two factors. First, the assessment landscape defined by the ACCE and HTA domains appears to have been sufficiently established, and methodological efforts have shifted from developing new frameworks to refining existing ones [52, 53]. Second, at least in the EU, the HTA collaboration has evolved from voluntary networks to a statutory framework, reducing the incentive to develop national disease‐ or technology‐specific assessment instruments in favor of more general frameworks [12]; this finding, coupled with the discontinuation of several earlier models, reinforces the view that overcoming methodological fragmentation remains a shared priority in the field [8, 50]. It is also comforting in light of the extreme methodological fragmentation we found: the frameworks identified varied considerably in structure and purpose and showed substantial inconsistency in addressing key assessment domains and components, confirming the absence of a gold standard for genome‐based technologies and thereby supporting the need to identify a universal framework to enhance their introduction in clinical practice [8].

Overall, frameworks based on the ACCE model were more focused on application and clinical aspects, but still seemed to lack some coverage in both the ‘personal and societal aspects’ and the ‘organizational aspects’ domain [7]. In other words, it appears that the numerous frameworks derived from the ACCE model as a mean to enhance its evaluation performance [5, 54] have improved its comprehensiveness to some extent, but not completely. However, ACCE's influence persists today, particularly through its institutionalization of concepts such as “clinical utility,” broadly defined as a test's ability to improve health outcomes [55], and “personal utility,” which refers to the test‐related subjective benefits [56]. Such aspects have recently been included in the notion of ‘genomic utility’ as an attempt to reflect the multi‐dimensional value of genomic applications [57], but this new concept is still evolving and definition and measurement consensus remains incomplete [52]. On the other hand, HTA‐inspired frameworks, while offering greater coverage for the organizational aspects, seemed to give less consideration to the application domain. In addition, similarly to the ACCE‐based frameworks, these models underestimated personal and societal aspects, features that are considered increasingly relevant in the evaluation of genetic and genomic testing [53]. Therefore, it does not surprise that the Sapienza framework, combining the ACCE and HTA models and derived from a literature review [7], offered substantial completeness.

However, we believe completeness is a necessary but insufficient criterion for an evaluation tool intended to serve as a standard, and the Sapienza framework still lacks validation and implementation, that is, widespread use in different context, as indicated by its original developers [40], and by only a few documented applications in practice [58, 59], a limitation that also affects other models identified in our analysis [23, 28, 32, 33, 42]. Moreover, it should be noted that the Sapienza framework is specifically tailored to genetic and genomic applications, but to facilitate policy decision‐making, evidence comparability is required, which in turn calls for evaluating emerging technologies within a unified framework and, consequently, avoiding technology‐specific assessment models [60]. These aspects together argue against its adoption as an evaluation framework for genetic and genomic applications.

In contrast, the HTA Core Model is a comprehensive and rigorous framework for evaluating health technologies that has undergone many updates and iterations over a decade of international collaboration [9, 10, 11]. Our analysis confirmed that it encompasses every component of the Sapienza framework, indicating that it can also serve as a solid basis for assessing genetic and genomic applications.

In addition, the experience of the HTA Core Model is particularly relevant, as the implementation of the EU HTA Regulation is only now beginning to translate this methodological investment into practice, with the first JCA published in June 2026 [61]. Although the scope of JCAs is expected to expand progressively until 2030, genetic and genomic applications largely remain outside the scope of the Regulation and will therefore continue to be assessed primarily at the national level within the EU [12]. This makes it particularly important that national HTA bodies do not adopt fragmented or divergent approaches to these technologies but instead apply a shared methodology that facilitates cross‐border assessment and comparison and promotes greater transparency in decision‐making. Therefore, an international effort is still needed to build on the established HTA methodology and strengthen it with considerations specific to genetic and genomic technologies.

A further advantage is that it could offer clear guidance to technology developers on the evidence expected to support evaluation. This is especially important in the field of genetic and genomic testing, where methodological heterogeneity and evidence gaps have been consistently identified as major obstacles to robust assessment [6] and, without rigorous assessment reports, evidence‐informed policy‐making becomes considerably more difficult. In addition, even though coverage was uneven, as the number of HTA issues varied largely across assessment domains, in our comparison every component identified in existing evaluation frameworks was mapped by specific Core Model issues, indicating extended coverage. Moreover, several components aligned with multiple HTA issues across different assessment domains, capturing the cross‐cutting nature of many aspects. This multidimensional interplay is particularly evident in the evaluation of genetic and genomic technologies, as genomic testing informs decisions and outcomes across the entire healthcare pathway [62, 63]. Despite complicating evidence search and synthesis during the evaluation process, this shift from test‐centric to program‐centric evaluation is essential [63], and the HTA Core Model seemed to sustain end‐to‐end pathway assessment. This is further confirmed by its particular attention to organizational aspects, including the resources needed to implement the program, any barrier, and associated costs – critical elements for policy decision‐making [64].

As for the other dimensions, we found that analytical and clinical testing performance were clearly addressed. Similarly, the clinical utility and downstream consequences were sufficiently represented, while the Model's completeness in investigating ethical, legal and social implications was remarkable, as the current landscape of HTA reports on genetic and genomic technologies tends to overlook these domains [6]. Considering all these aspects, we deemed the HTA Core Model to be broadly suitable for the evaluation of genomic technologies, as all core questions were captured. However, although not explicitly articulated in any retrieved framework, we also noted that several refinements could be applied to the Model to better address genomic‐specific features: for example, there are certain testing aspects (e.g., variant interpretation, gene penetrance, genetically transitional diseases) [65], challenges related to novel technologies (e.g., polygenic risk scores) [66, 67] and to potential privacy risks posed by storage and sharing of genomic data (e.g., identification and genotype inference) [68], that may be not sufficiently detailed in the current version of the HTA Core Model. In this regard, we believe that the fast‐paced development and availability of new health technologies, together with the continuous emergence of technology‐specific issues, pose challenges that any evaluation process will inevitably face, regardless of the framework adopted. These challenges could be faced through the adoption of new methods for both generating evidence (e.g., adaptive, umbrella, and basket trials) [69, 70, 71], which, however, only marginally fall within the scope of the HTA process itself, and synthesizing it (e.g., the use of large language models to support systematic reviews) [72], although most of these concepts are still at an early stage of development and implementation. Moreover, although the Model addresses incidental findings, longitudinal reinterpretation and practical aspects of recontacting patients are not clearly reported. Another example is cascade testing: while legal and ethical hooks for relatives exist, the Model could benefit from more precise prompts on resources needed and service reconfiguration for a real‐world implementation of cascade pathways. For these reasons, while we showed that the HTA Core Model could provide a sufficiently complete architecture for evaluating genetic and genomic technologies, a structured and systematic approach should be conducted to operationalize it with specific items whenever there are issues deemed silent or partially unaddressed. Given its hierarchical structure, reporting format, which presents information as question‐answer pairs [19], and capacity for expansion and revision [73], integrating refinements for unaddressed or emerging issues would not require structural changes to the Model. By contrast, undertaking this process would preserve the advantages of a single, methodologically robust evaluation framework, capable of effectively assessing the specificities of genomic technologies while maintaining a common HTA approach.

This because the HTA process, although notably time‐consuming [74], is widely regarded as the gold standard for health technology evaluation [8, 75]. Nevertheless, as aforementioned, the evaluation of genetic and genomic applications is often constrained by evidence gaps [6], particularly for complex technologies such as whole‐genome and whole‐exome sequencing [76]. In such circumstances, where strict adherence to methodological rigor could slow the introduction of these technologies into clinical practice, the initial use of alternative assessment approaches, in lieu of full HTA evaluations, may be justified. For example, the HTA Core Model rapid relative effectiveness assessment (REA) approach [77] focuses on the first four HTA Core Model domains (i.e., technical characteristics of technology, current use of technology, clinical effectiveness, and safety) which are also the domains addressed in JCAs under the EU HTA Regulation [12]. In our view, once minimum thresholds of effectiveness and safety are met, an initially narrower appraisal of a specific technology use case (e.g., the detection of genetic or genomic alterations for the diagnosis of rare diseases in pediatric settings) through REA methods could permit conditional introduction into clinical practice, either within research projects or, alternatively, under financial risk‐sharing arrangements [78]. This pathway would enable prospective generation of evidence for the remaining domains, without making adoption contingent on the prior availability of exhaustive and comprehensive evidence, a requirement that may take many years to fulfill given the numerous challenges reported in evidence generation [6].

Some limitations must be acknowledged. First, because several frameworks identified in earlier systematic reviews were not retrievable, we cannot exclude to have missed a few domains or components; however, if so, it likely pertained to frameworks that were subsequently discontinued, probably indicating low relevance. Second, it is possible that the comparison process was influenced by the individual interpretations. Nevertheless, the substantial agreement achieved between the two reviewers conducting the mapping likely minimized this limitation. Disagreements, however, were more frequent in the clinical, organizational, health condition and technology domains, confirming that certain issues may include aspects that span multiple assessment components. Despite demonstrating full coverage in all identified assessment components, the HTA Core Model (version 3.0) may need some refinements. Thanks to its flexible structure, some further research should be done to integrate it with genomics‐specific items to maximize its completeness in the evaluation of genetic and genomic applications. Finally, eligible documents were restricted to those in English or Italian. Although this allowed us to identify frameworks by most relevant institutions, which use English as their working language, assessment frameworks developed at national level and disseminated only in other languages may have been missed.

## 6 Conclusions

To end methodological fragmentation and facilitate the implementation process, consensus must be built around a common, standardized framework for the evaluation of genetic and genomic technologies. Compared to the previous systematic reviews, this update did not identify new frameworks, but applied a more detailed and standardized extraction process, enabling the identification of not only the assessment domains but also the specific components considered by each framework. This allowed, to the greatest extent possible, a detailed comparison between frameworks tailored to genetic and genomic applications and the ‘general‐purpose’ HTA Core Model (version 3.0), showing substantial concordance, and, thus, indicating it as an optimal candidate to serve as a universal framework capable of effectively assessing genetic and genomic applications while preserving the overarching HTA approach. Future research should therefore explore optimal ways of including emerging genomic‐specific aspects into the HTA Core Model to further enhance its comprehensiveness and applicability.

## Author Contributions

**Valentina Baccolini:** conceptualization, formal analysis, writing – original draft preparation. **Antonio Sciurti:** conceptualization, data curation, formal analysis, writing – original draft preparation. **Erica Pitini:** conceptualization, writing – review and editing. **Leonardo Maria Siena:** data curation, writing – review and editing. **Arianna Anniballo:** data curation. **Anna Ewa Kaminska:** data curation. **Andrea Pistollato:** data curation. **Romina Pausilli:** data curation. **Chiara Tommasino:** data curation. **Carolina Marzuillo:** writing – review and editing. **Corrado De Vito:** writing – review and editing. **Giuseppe La Torre:** writing – review and editing. **Giuseppe Migliara:** conceptualization, formal analysis, writing – review and editing, supervision. **Paolo Villari:** writing – review and editing, funding acquisition, supervision.

## Disclosure

All authors have read and approved the final version of the manuscript. The lead authors, Valentina Baccolini and Antonio Sciurti, had full access to all of the data in this study and take complete responsibility for the integrity of the data and the accuracy of the data analysis.

## Conflicts of Interest

The authors declare no conflicts of interest.

## Transparency Statement

The lead authors, Valentina Baccolini and Antonio Sciurti, affirm that this manuscript is an honest, accurate and transparent account of the study being reported; that no important aspects of the study have been omitted; and that any discrepancies from the study as planned have been explained.

## Supporting information


Supporting File

## Data Availability

The authors confirm that the data supporting the findings of this study are available within the article and its Supporting Information.
