# Supplementary material for: From Fragmentation to Standardization: A Systematic Review Update and Critical Appraisal of Genetic and Genomic Testing Assessment Frameworks and Their Alignment With the HTA Core Model
Source: Health Sci Rep. 2026 Oct 28;9(11):e73446. doi: 10.1002/hsr2.73446 (PMC13715073; doi:10.1002/hsr2.73446)
Supplement: Supplementary file 1 [file HSR2-9-e73446-s001.docx]

**Supporting Table 1.** Search strategies used in the systematic review by database.

| **Database** | **Search strategy** | |
| --- | --- | --- |
| PubMed | ((((((“genetic testing”[Title/Abstract] OR “genetic test”[Title/Abstract] OR “genetic tests”[Title/Abstract] OR “genetic technology”[Title/Abstract] OR “genetic technologies”[Title/Abstract] OR “genetic panel”[Title/Abstract] OR “genetic panels”[Title/Abstract] OR “gene panel”[Title/Abstract] OR “gene panels”[Title/Abstract] OR “genetic profiling”[Title/Abstract] OR “genetic application”[Title/Abstract] OR “genetic applications”[Title/Abstract] OR “genetic screening”[Title/Abstract] OR “genomic testing”[Title/Abstract] OR “genomic test”[Title/Abstract] OR “genomic tests”[Title/Abstract] OR “genomic technology”[Title/Abstract] OR “genomic technologies”[Title/Abstract] OR “genomic panel”[Title/Abstract] OR “genomic panels”[Title/Abstract] OR “genome panel”[Title/Abstract] OR “genome panels”[Title/Abstract] OR “genomic profiling”[Title/Abstract] OR “genomic application”[Title/Abstract] OR “genomic applications”[Title/Abstract] OR “genomic screening”[Title/Abstract] OR “next generation sequencing testing”[Title/Abstract] OR “NGS testing”[Title/Abstract] OR “next generation sequencing test”[Title/Abstract] OR “next generation sequencing tests”[Title/Abstract] OR “NGS test”[Title/Abstract] OR “NGS tests”[Title/Abstract] OR “next generation sequencing technology”[Title/Abstract] OR “next generation sequencing technologies”[Title/Abstract] OR “NGS technology”[Title/Abstract] OR “NGS technologies”[Title/Abstract] OR “next generation sequencing panel”[Title/Abstract] OR “next generation sequencing panels”[Title/Abstract] OR “NGS panel”[Title/Abstract] OR “NGS panels”[Title/Abstract] OR “next generation sequencing profiling”[Title/Abstract] OR “NGS profiling”[Title/Abstract] OR “next generation sequencing application”[Title/Abstract] OR “next generation sequencing applications”[Title/Abstract] OR “NGS application”[Title/Abstract] OR “NGS applications”[Title/Abstract] OR “next generation sequencing screening”[Title/Abstract] OR “NGS screening”[Title/Abstract] OR “pharmacogenetic testing”[Title/Abstract] OR “pharmacogenetic test”[Title/Abstract] OR “pharmacogenetic tests”[Title/Abstract] OR “pharmacogenetic technology”[Title/Abstract] OR “pharmacogenetic technologies”[Title/Abstract] OR “pharmacogenetic panel”[Title/Abstract] OR “pharmacogenetic panels”[Title/Abstract] OR “pharmacogenetic profiling”[Title/Abstract] OR “pharmacogenetic application”[Title/Abstract] OR “pharmacogenetic applications”[Title/Abstract] OR “pharmacogenetic screening”[Title/Abstract] OR “pharmacogenomic testing”[Title/Abstract] OR “pharmacogenomic test”[Title/Abstract] OR “pharmacogenomic tests”[Title/Abstract] OR “pharmacogenomic technology”[Title/Abstract] OR “pharmacogenomic technologies”[Title/Abstract] OR “pharmacogenomic panel”[Title/Abstract] OR “pharmacogenomic panels”[Title/Abstract] OR “pharmacogenomic profiling”[Title/Abstract] OR “pharmacogenomic application”[Title/Abstract] OR “pharmacogenomic applications”[Title/Abstract] OR “pharmacogenomic screening”[Title/Abstract] OR “molecular testing”[Title/Abstract] OR “molecular test”[Title/Abstract] OR “molecular tests”[Title/Abstract] OR “molecular technology”[Title/Abstract] OR “molecular technologies”[Title/Abstract] OR “molecular panel”[Title/Abstract] OR “molecular panels”[Title/Abstract] OR “molecular profiling”[Title/Abstract] OR “molecular application”[Title/Abstract] OR “molecular applications”[Title/Abstract] OR “molecular screening”[Title/Abstract] OR “genetic predisposition testing”[Title/Abstract] OR “predictive testing”[Title/Abstract]))) AND ((evaluation[Title/Abstract] OR evaluations[Title/Abstract] OR evaluate[Title/Abstract] OR evaluating[Title/Abstract] OR evaluated[Title/Abstract] OR assessment[Title/Abstract] OR assessments[Title/Abstract] OR assess[Title/Abstract] OR assessing[Title/Abstract] OR assessed[Title/Abstract] OR “health technology assessment”[Title/Abstract] OR “HTA”[Title/Abstract] OR “evidence review”[Title/Abstract] OR “evidence reviews”[Title/Abstract]))) AND ((framework[Title/Abstract] OR tool[Title/Abstract] OR model[Title/Abstract] OR process[Title/Abstract] OR method[Title/Abstract] OR methodology[Title/Abstract] OR checklist[Title/Abstract] OR experience[Title/Abstract] OR domains[Title/Abstract] OR criteria[Title/Abstract] OR dimensions[Title/Abstract] OR questions[Title/Abstract] OR principles[Title/Abstract] OR “analytic validity”[Title/Abstract] OR “clinical validity”[Title/Abstract] OR “clinical utility”[Title/Abstract] OR efficacy[Title/Abstract] OR effectiveness[Title/Abstract] OR economic[Title/Abstract] OR “evidence based”[Title/Abstract])))) OR ((((“Genetic Testing”[Mesh]) OR “Pharmacogenomic Testing”[Mesh])) AND ((“Technology Assessment, Biomedical”[Mesh]) OR “Diagnostic Test Approval”[Mesh]))  **Date range:** October 10^th^ 2020 – May 7^th^ 2025 | |
| Scopus | TITLE-ABS-KEY (“genetic testing” OR “genetic test” OR “genetic tests” OR “genetic technology” OR “genetic technologies” OR “genetic panel” OR “genetic panels” OR “gene panel” OR “gene panels” OR “genetic profiling” OR “genetic application” OR “genetic applications” OR “genetic screening” OR “genomic testing” OR “genomic test” OR “genomic tests” OR “genomic technology” OR “genomic technologies” OR “genomic panel” OR “genomic panels” OR “genome panel” OR “genome panels” OR “genomic profiling” OR “genomic application” OR “genomic applications” OR “genomic screening” OR “next generation sequencing testing” OR “NGS testing” OR “next generation sequencing test” OR “next generation sequencing tests” OR “NGS test” OR “NGS tests” OR “next generation sequencing technology” OR “next generation sequencing technologies” OR “NGS technology” OR “NGS technologies” OR “next generation sequencing panel” OR “next generation sequencing panels” OR “NGS panel” OR “NGS panels” OR “next generation sequencing profiling” OR “NGS profiling” OR “next generation sequencing application” OR “next generation sequencing applications” OR “NGS application” OR “NGS applications” OR “next generation sequencing screening” OR “NGS screening” OR “pharmacogenetic testing” OR “pharmacogenetic test” OR “pharmacogenetic tests” OR “pharmacogenetic technology” OR “pharmacogenetic technologies” OR “pharmacogenetic panel” OR “pharmacogenetic panels” OR “pharmacogenetic profiling” OR “pharmacogenetic application” OR “pharmacogenetic applications” OR “pharmacogenetic screening” OR “pharmacogenomic testing” OR “pharmacogenomic test” OR “pharmacogenomic tests” OR “pharmacogenomic technology” OR “pharmacogenomic technologies” OR “pharmacogenomic panel” OR “pharmacogenomic panels” OR “pharmacogenomic profiling” OR “pharmacogenomic application” OR “pharmacogenomic applications” OR “pharmacogenomic screening” OR “molecular testing” OR “molecular test” OR “molecular tests” OR “molecular technology” OR “molecular technologies” OR “molecular panel” OR “molecular panels” OR “molecular profiling” OR “molecular application” OR “molecular applications” OR “molecular screening” OR “genetic predisposition testing” OR “predictive testing”) AND TITLE-ABS-KEY(evaluation OR evaluations OR evaluate OR evaluating OR evaluated OR assessment OR assessments OR assess OR assessing OR assessed OR “health technology assessment” OR “HTA” OR “evidence review” OR “evidence reviews” ) AND TITLE-ABS-KEY ( framework OR tool OR model OR process OR method OR methodology OR checklist OR experience OR domains OR criteria OR dimensions OR questions OR principles OR “analytic validity” OR “clinical validity” OR “clinical utility” OR efficacy OR effectiveness OR economic OR “evidence based”) **AND** **ORIG-LOAD-DATE AFT 20201001** | |
| Web of Science | TS=(“genetic testing” OR “genetic test” OR “genetic tests” OR “genetic technology” OR “genetic technologies” OR “genetic panel” OR “genetic panels” OR “gene panel” OR “gene panels” OR “genetic profiling” OR “genetic application” OR “genetic applications” OR “genetic screening” OR “genomic testing” OR “genomic test” OR “genomic tests” OR “genomic technology” OR “genomic technologies” OR “genomic panel” OR “genomic panels” OR “genome panel” OR “genome panels” OR “genomic profiling” OR “genomic application” OR “genomic applications” OR “genomic screening” OR “next generation sequencing testing” OR “NGS testing” OR “next generation sequencing test” OR “next generation sequencing tests” OR “NGS test” OR “NGS tests” OR “next generation sequencing technology” OR “next generation sequencing technologies” OR “NGS technology” OR “NGS technologies” OR “next generation sequencing panel” OR “next generation sequencing panels” OR “NGS panel” OR “NGS panels” OR “next generation sequencing profiling” OR “NGS profiling” OR “next generation sequencing application” OR “next generation sequencing applications” OR “NGS application” OR “NGS applications” OR “next generation sequencing screening” OR “NGS screening” OR “pharmacogenetic testing” OR “pharmacogenetic test” OR “pharmacogenetic tests” OR “pharmacogenetic technology” OR “pharmacogenetic technologies” OR “pharmacogenetic panel” OR “pharmacogenetic panels” OR “pharmacogenetic profiling” OR “pharmacogenetic application” OR “pharmacogenetic applications” OR “pharmacogenetic screening” OR “pharmacogenomic testing” OR “pharmacogenomic test” OR “pharmacogenomic tests” OR “pharmacogenomic technology” OR “pharmacogenomic technologies” OR “pharmacogenomic panel” OR “pharmacogenomic panels” OR “pharmacogenomic profiling” OR “pharmacogenomic application” OR “pharmacogenomic applications” OR “pharmacogenomic screening” OR “molecular testing” OR “molecular test” OR “molecular tests” OR “molecular technology” OR “molecular technologies” OR “molecular panel” OR “molecular panels” OR “molecular profiling” OR “molecular application” OR “molecular applications” OR “molecular screening” OR “genetic predisposition testing” OR “predictive testing”) AND TS=(evaluation OR evaluations OR evaluate OR evaluating OR evaluated OR assessment OR assessments OR assess OR assessing OR “health technology assessment” OR assessed OR “HTA” OR “evidence review” OR “evidence reviews”) AND TS= (framework OR tool OR model OR process OR method OR methodology OR checklist OR experience OR domains OR criteria OR dimensions OR questions OR principles OR “analytic validity” OR “clinical validity” OR “clinical utility” OR efficacy OR effectiveness OR economic OR “evidence based”)  **Date range:** October 10^th^ 2020 – May 7^th^ 2025 | |
| Google Scholar | Search A | “genetic test” OR “genetic tests” OR “genomic test” OR “genomic tests” OR “health technology assessment”  **Date range:** 2020 –2025  **Filters:** no citations, no patents |
|  | Search B | “NGS test” OR “NGS tests” OR “NGS panel” OR “NGS panels” OR “next generation sequencing test” OR “next generation sequencing tests” OR “next generation sequencing panel” OR “next generation sequencing panels” OR “health technology assessment”  **Date range:** 2020 –2025  **Filters:** no citations, no patents |

**Supporting Table 2.** Frameworks reporting specific assessment component, by reference framework and assessment domain (N=24).

| **Domain** | **Component** | **ACCE only (N=12)** | | **HTA only (N=4)** | | **ACCE + HTA (N=1)** | | **Other framework (N=7)** | |
| --- | --- | --- | --- | --- | --- | --- | --- | --- | --- |
|  |  | **N (%)** | **Framework** | **N (%)** | **Framework** | **N (%)** | **Framework** | **N (%)** | **Framework** |
| **Disease/condition of interest** | **Epidemiology** | 11 (91.7) | Expanded ACCE; ACHDNC; CAT; Rapid ACCE; ACCE; Andalusian; Clinical Utility Card; GETT; EGAPP; UKGTN Gene Dossier; Complex Diseases | 3 (75.0) | McMaster Model; Framework for evaluating co-dependent technologies for a reimbursement decision; PACNPGT | 1 (100.0) | Sapienza | 4 (57.1) | Genetic Screening; Population screening for adult-onset conditions; Crossroads 99; SynFRAME |
|  | **Etiopathogenesis** | 5 (41.7) | Expanded ACCE; Rapid ACCE; Clinical Utility Card; GETT; Complex Diseases | 1 (25.0) | Framework for evaluating co-dependent technologies for a reimbursement decision | 1 (100.0) | Sapienza | 2 (28.6) | Genetic Screening; Population screening for adult-onset conditions |
|  | **Natural history** | 6 (50.0) | Expanded ACCE; ACHDNC; ACCE; Andalusian; GETT; EGAPP | 1 (25.0) | PACNPGT | 1 (100.0) | Sapienza | 4 (57.1) | Genetic Screening; Population screening for adult-onset conditions; Crossroads 99; SynFRAME |
|  | **Current management** | 7 (58.3) | Expanded ACCE; ACHDNC; CAT; Andalusian; Clinical Utility Card; GETT; EGAPP | 2 (50.0) | Framework for evaluating co-dependent technologies for a reimbursement decision; PACNPGT | 1 (100.0) | Sapienza | 5 (71.4) | Population screening for adult-onset conditions; SynFRAME; Practical framework; Evidence framework for genetic testing; ECRI |
| **Genetic/genomic application** | **Classification** | 11 (91.7) | Expanded ACCE; ACHDNC; CAT; Six-part framework; Rapid ACCE; ACCE; Andalusian; Clinical Utility Card; GETT; EGAPP; Complex Diseases | 3 (75.0) | McMaster Model; Framework for evaluating co-dependent technologies for a reimbursement decision; PACNPGT | 1 (100.0) | Sapienza | 6 (85.7) | Genetic Screening; Population screening for adult-onset conditions; Crossroads 99; SynFRAME; Practical framework; ECRI |
|  | **Type of technology** | 8 (66.7) | Expanded ACCE; Rapid ACCE; ACCE; Andalusian; Clinical Utility Card; GETT; EGAPP; Complex Diseases | 2 (50.0) | Framework for evaluating co-dependent technologies for a reimbursement decision; PACNPGT | 1 (100.0) | Sapienza | 2 (28.6) | Genetic Screening; ECRI |
|  | **Analytical accuracy** | 10 (83.3) | Expanded ACCE; ACHDNC; Six-part framework; Rapid ACCE; ACCE; Andalusian; Clinical Utility Card; GETT; EGAPP; UKGTN Gene Dossier | 3 (75.0) | McMaster Model; Framework for evaluating co-dependent technologies for a reimbursement decision; PACNPGT | 1 (100.0) | Sapienza | 4 (57.1) | Population screening for adult-onset conditions; SynFRAME; Practical framework; ECRI |
| **Clinical aspects** | **Purpose** | 12 (100.0) | Expanded ACCE; ACHDNC; CAT; Six-part framework; Rapid ACCE; ACCE; Andalusian; Clinical Utility Card; GETT; EGAPP; UKGTN Gene Dossier; Complex Diseases | 3 (75.0) | McMaster Model; Framework for evaluating co-dependent technologies for a reimbursement decision; PACNPGT | 1 (100.0) | Sapienza | 7 (100.0) | Genetic Screening; Population screening for adult-onset conditions; Crossroads 99; SynFRAME; Practical framework; Evidence framework for genetic testing; ECRI |
|  | **Target population** | 11 (91.7) | Expanded ACCE; CAT; Six-part framework; Rapid ACCE; ACCE; Andalusian; Clinical Utility Card; GETT; EGAPP; UKGTN Gene Dossier; Complex Diseases | 4 (100.0) | McMaster Model; HTA Personalized Health Care; Framework for evaluating co-dependent technologies for a reimbursement decision; PACNPGT | 1 (100.0) | Sapienza | 7 (100.0) | Genetic Screening; Population screening for adult-onset conditions; Crossroads 99; SynFRAME; Practical framework; Evidence framework for genetic testing; ECRI |
|  | **Clinical setting** | 9 (75.0) | Expanded ACCE; Six-part framework; Rapid ACCE; ACCE; Andalusian; Clinical Utility Card; EGAPP; UKGTN Gene Dossier; Complex Diseases | 2 (50.0) | McMaster Model; Framework for evaluating co-dependent technologies for a reimbursement decision | 1 (100.0) | Sapienza | 6 (85.7) | Genetic Screening; Population screening for adult-onset conditions; Crossroads 99; Practical framework; Evidence framework for genetic testing; ECRI |
|  | **Post-test intervention** | 10 (83.3) | Expanded ACCE; ACHDNC; CAT; Six-part framework; Rapid ACCE; ACCE; Clinical Utility Card; GETT; EGAPP; Complex Diseases | 4 (100.0) | McMaster Model; HTA Personalized Health Care; Framework for evaluating co-dependent technologies for a reimbursement decision; PACNPGT | 1 (100.0) | Sapienza | 6 (85.7) | Genetic Screening; Population screening for adult-onset conditions; Crossroads 99; SynFRAME; Practical framework; ECRI |
|  | **Post-test clinical pathways** | 8 (66.7) | ACHDNC; CAT; Six-part framework; Rapid ACCE; ACCE; GETT; EGAPP; Complex Diseases | 3 (75.0) | McMaster Model; Framework for evaluating co-dependent technologies for a reimbursement decision; PACNPGT | 1 (100.0) | Sapienza | 6 (85.7) | Genetic Screening; Population screening for adult-onset conditions; Crossroads 99; SynFRAME; Practical framework; ECRI |
|  | **Efficacy** | 10 (83.3) | Expanded ACCE; ACHDNC; CAT; Six-part framework; Rapid ACCE; ACCE; Andalusian; Clinical Utility Card; EGAPP; Complex Diseases | 2 (50.0) | McMaster Model; Framework for evaluating co-dependent technologies for a reimbursement decision | 1 (100.0) | Sapienza | 5 (71.4) | Genetic Screening; Population screening for adult-onset conditions; SynFRAME; Practical framework; ECRI |
|  | **Effectiveness** | 11 (91.7) | Expanded ACCE; ACHDNC; CAT; Six-part framework; Rapid ACCE; ACCE; Andalusian; Clinical Utility Card; GETT; EGAPP; Complex Diseases | 4 (100.0) | McMaster Model; HTA Personalized Health Care; Framework for evaluating co-dependent technologies for a reimbursement decision; PACNPGT | 1 (100.0) | Sapienza | 6 (85.7) | Genetic Screening; Population screening for adult-onset conditions; Crossroads 99; SynFRAME; Practical framework; ECRI |
|  | **Safety** | 7 (58.3) | Expanded ACCE; ACHDNC; Rapid ACCE; ACCE; Andalusian; GETT; EGAPP | 4 (100.0) | McMaster Model; HTA Personalized Health Care; Framework for evaluating co-dependent technologies for a reimbursement decision; PACNPGT | 1 (100.0) | Sapienza | 6 (85.7) | Genetic Screening; Population screening for adult-onset conditions; Crossroads 99; Practical framework; Evidence framework for genetic testing; ECRI |
|  | **Patients reported outcomes** | 2 (16.7) | ACHDNC; EGAPP | 1 (25.0) | McMaster Model | 1 (100.0) | Sapienza | 2 (28.6) | Genetic Screening; Practical framework |
|  | **Clinical accuracy** | 11 (91.7) | Expanded ACCE; ACHDNC; CAT; Rapid ACCE; ACCE; Andalusian; Clinical Utility Card; GETT; EGAPP; UKGTN Gene Dossier; Complex Diseases | 3 (75.0) | McMaster Model; Framework for evaluating co-dependent technologies for a reimbursement decision; PACNPGT | 1 (100.0) | Sapienza | 3 (42.9) | Population screening for adult-onset conditions; Crossroads 99; SynFRAME |
| **Personal and societal aspects** | **Non-health-related outcomes** | 4 (33.3) | ACHDNC; Six-part framework; ACCE; Complex Diseases | 3 (75.0) | McMaster Model; Framework for evaluating co-dependent technologies for a reimbursement decision; PACNPGT | 1 (100.0) | Sapienza | 5 (71.4) | Genetic Screening; Crossroads 99; SynFRAME; Practical framework; ECRI |
|  | **Ethical implications** | 9 (75.0) | Expanded ACCE; ACHDNC; Rapid ACCE; ACCE; Andalusian; GETT; EGAPP; UKGTN Gene Dossier; Complex Diseases | 3 (75.0) | McMaster Model; HTA Personalized Health Care; PACNPGT | 1 (100.0) | Sapienza | 6 (85.7) | Genetic Screening; Population screening for adult-onset conditions; Crossroads 99; SynFRAME; Practical framework; ECRI |
|  | **Legal implications** | 7 (58.3) | Expanded ACCE; ACHDNC; Rapid ACCE; ACCE; GETT; EGAPP; Complex Diseases | 3 (75.0) | McMaster Model; HTA Personalized Health Care; PACNPGT | 1 (100.0) | Sapienza | 6 (85.7) | Genetic Screening; Population screening for adult-onset conditions; Crossroads 99; SynFRAME; Practical framework; ECRI |
|  | **Social implications** | 9 (75.0) | Expanded ACCE; ACHDNC; Rapid ACCE; ACCE; Andalusian; GETT; EGAPP; UKGTN Gene Dossier; Complex Diseases | 3 (75.0) | McMaster Model; HTA Personalized Health Care; PACNPGT | 1 (100.0) | Sapienza | 7 (100.0) | Genetic Screening; Population screening for adult-onset conditions; Crossroads 99; SynFRAME; Practical framework; Evidence framework for genetic testing; ECRI |
| **Organizational aspects** | **Burden of disease** | 9 (75.0) | Expanded ACCE; ACHDNC; Rapid ACCE; ACCE; Andalusian; Clinical Utility Card; GETT; EGAPP; Complex Diseases | 3 (75.0) | McMaster Model; Framework for evaluating co-dependent technologies for a reimbursement decision; PACNPGT | 1 (100.0) | Sapienza | 4 (57.1) | Genetic Screening; Population screening for adult-onset conditions; Crossroads 99; SynFRAME |
|  | **Professional resources needs** | 4 (33.3) | ACCE; Andalusian; GETT; Complex Diseases | 3 (75.0) | McMaster Model; HTA Personalized Health Care; PACNPGT | 1 (100.0) | Sapienza | 4 (57.1) | Genetic Screening; Population screening for adult-onset conditions; Crossroads 99; ECRI |
|  | **Material resources needs** | 7 (58.3) | Expanded ACCE; Six-part framework; ACCE; Andalusian; Clinical Utility Card; GETT; Complex Diseases | 3 (75.0) | McMaster Model; Framework for evaluating co-dependent technologies for a reimbursement decision; PACNPGT | 1 (100.0) | Sapienza | 4 (57.1) | Genetic Screening; Population screening for adult-onset conditions; Crossroads 99; ECRI |
|  | **Financial resources needs** | 9 (75.0) | Expanded ACCE; CAT; Six-part framework; ACCE; Andalusian; Clinical Utility Card; GETT; UKGTN Gene Dossier; Complex Diseases | 4 (100.0) | McMaster Model; HTA Personalized Health Care; Framework for evaluating co-dependent technologies for a reimbursement decision; PACNPGT | 1 (100.0) | Sapienza | 7 (100.0) | Genetic Screening; Population screening for adult-onset conditions; Crossroads 99; SynFRAME; Practical framework; Evidence framework for genetic testing; ECRI |
|  | **Economic evaluation** | 11 (91.7) | Expanded ACCE; ACHDNC; CAT; Six-part framework; Rapid ACCE; ACCE; Andalusian; Clinical Utility Card; GETT; EGAPP; Complex Diseases | 4 (100.0) | McMaster Model; HTA Personalized Health Care; Framework for evaluating co-dependent technologies for a reimbursement decision; PACNPGT | 1 (100.0) | Sapienza | 6 (85.7) | Genetic Screening; Population screening for adult-onset conditions; Crossroads 99; SynFRAME; Practical framework; Evidence framework for genetic testing |
|  | **Equity in access** | 5 (41.7) | Expanded ACCE; Rapid ACCE; Andalusian; GETT; Complex Diseases | 3 (75.0) | McMaster Model; HTA Personalized Health Care; PACNPGT | 1 (100.0) | Sapienza | 4 (57.1) | Genetic Screening; Population screening for adult-onset conditions; Crossroads 99; SynFRAME |
|  | **Barriers to implementation** | 2 (16.7) | ACCE; GETT | 1 (25.0) | HTA Personalized Health Care | 1 (100.0) | Sapienza | 1 (14.3) | Genetic Screening |
| **Reporting aspects** | **Assessment of quality of the evidence** | 6 (50.0) | ACHDNC; CAT; Rapid ACCE; Andalusian; Clinical Utility Card; EGAPP | 4 (100.0) | McMaster Model; HTA Personalized Health Care; Framework for evaluating co-dependent technologies for a reimbursement decision; PACNPGT | 1 (100.0) | Sapienza | 3 (42.9) | SynFRAME; Evidence framework for genetic testing; ECRI |
|  | **Reporting of evidence gaps/research priorities** | 7 (58.3) | Expanded ACCE; ACHDNC; Rapid ACCE; ACCE; Andalusian; EGAPP; UKGTN Gene Dossier | 2 (50.0) | HTA Personalized Health Care; PACNPGT | 1 (100.0) | Sapienza | 6 (85.7) | Genetic Screening; Crossroads 99; SynFRAME; Practical framework; Evidence framework for genetic testing; ECRI |
| ACCE (Analytic validity, Clinical validity, Clinical utility, Ethical, legal and Social Implications) (1,2); ACHDNC (Advisory Committee on Heritable Disorders in Newborns and Children) (3); Andalusian (4); CAT (Companion Tests Assessment Tool) (5); Clinical Utility Card (6); Complex Diseases (7); Crossroads 99 (8); ECRI (Emergency Care Research Institute) (9); EGAPP (Evaluation of Genomic Applications in Practice and Prevention) (10,11); Evidence framework for genetic testing (12); Expanded ACCE (13); Framework for co-dependent technologies (14); Genetic Screening (15,16); GETT (Genetic testing Evidence Tracking Tool) (17); HTA (Health Technology Assessment) Personalized Health Care (18); McMaster Model (19); PACNPGT (Provincial Advisory Committee on New Predictive Genetic Technologies) (20); Population screening for adult-onset conditions (21); Practical framework (22);Rapid ACCE (23,24); Sapienza (25); Six-part framework (26); SynFRAME (27); UKGTN (UK Genetic Testing Network) gene dossier (28,29). | | | | | | | | | |

**Supporting Table 2 References**

1.	Centers for Disease Control and prevention. First ACCE Review: Population-based Prenatal Screening for Cystic Fibrosis via Carrier Testing [Internet]. 2002. Available from: archive.cdc.gov/www_cdc_gov/genomics/gtesting/acce/acce.htm

2.	Haddow JE, Palomaki GE. ACCE: a model process for evaluating data on emerging genetic tests. In: Khoury M, Little J, Burke W, editors. Human Genome Epidemiology : A Scientific Foundation for Using Genetic Information to Improve Health and Prevent Disease. Oxford, UK: Oxford University Press; 2004. p. 217–33.

3.	Calonge N, Green NS, Rinaldo P. Committee report: method for evaluating conditions nominated for population-based screening of newborns and children. Genetics in Medicine. 2010;3:153–9.

4.	Márquez Calderón S, Briones Pérez de la Blanca E. Framework for the assessment of genetic testing in the Andalusian Public Health System [Internet]. Andalusian Agency for Health Technology Assessment; 2006. Available from: aetsa.org/publicacion/marco-de-evaluacion-de-pruebas-geneticas-en-el-sistema-sanitario-publico-de-andalucia

5.	Canestaro WJ, Pritchard DE, Garrison LP, Dubois R, Veenstra DL. Improving the efficiency and quality of the value assessment process for companion diagnostic tests: The Companion test Assessment Tool (CAT). Journal of Managed Care & Specialty Pharmacy. 2015;21:700–12.

6.	Medical Service Advisory Committee. Clinical Utility Card for heritable mutations which increase risk in [disease area]. [Internet]. 2016. Available from: msac.gov.au/internet/msac/publishing.nsf/Content/9C7DCF1C2DD56CBECA25801000123C32/$File/Glossary-CUC-proforma.pdf

7.	Wright CF, Kroese M. Evaluation of genetic tests for susceptibility to common complex diseases: why, when and how? Human Genetics. 2010;127:125–34.

8.	Goel V. Appraising organised screening programmes for testing for genetic susceptibility to cancer. British Medical Journal. 2001;7295:1174–8.

9.	Sun F, Bruening W, Erinoff E, Schoelles KM. ECRI Institute Evidence-based Practice Center. Addressing challenges in genetic test evaluation. Evaluation frameworks and assessment of analytic validity. Agency for Healthcare Research and Quality [Internet]. 2011; Available from: ncbi.nlm.nih.gov/books/NBK56750

10.	Teutsch SM, Bradley LA, Palomaki GE. The evaluation of genomic applications in practice and prevention (EGAPP) initiative: methods of the EGAPP Working Group. Genetics in Medicine. 2009;1:3–14.

11.	Veenstra DL, Piper M, Haddow JE. Improving the efficiency and relevance of evidence-based recommendations in the era of whole-genome sequencing: an EGAPP methods update. Genetics in Medicine. 2013;1:14–24.

12.	National Academies of Sciences, Engineering, and Medicine. An evidence framework for genetic testing [Internet]. The National Academies Press; 2017. Available from: nap.nationalacademies.org/catalog/24632/an-evidence-framework-for-genetic-testing

13.	Burke W, Zimmern R. Moving beyond ACCE: an expanded framework for genetic test evaluation [Internet]. PHG Foundation; 2007. Available from: phgfoundation.org/publications/reports/moving-beyond-acce-an-expanded-framework-for-genetic-test-evaluation

14.	Merlin T, Farah C, Schubert C, Mitchell A, Hiller JE, Ryan P. Assessing personalized medicines in Australia: a national framework for reviewing codependent technologies. Medical Decision Making. 2013;3:333–42.

15.	Andermann A, Blancquaert I, Déry V. Genetic screening: a conceptual framework for programs and policy-making. Journal of Health Services Research & Policy. 2010;2:90–7.

16.	Andermann A, Blancquaert I, Beauchamp S, Costea I. Guiding policy decisions for genetic screening: developing a systematic and transparent approach. Public Health Genomics. 2011;1:9–16.

17.	Rousseau F, Lindsay C, Charland M. Development and description of GETT: a genetic testing evidence tracking tool. Clinical Chemistry and Laboratory Medicine. 2010;10:1397–407.

18.	Gutiérrez-Ibarluzea I. Personalised Health Care, the need for reassessment. A HTA perspective far beyond cost-effectiveness. Italian Journal of Public Health [Internet]. 2012; Available from: https://doi.org/10.2427/8653

19.	Giacomini M, Miller F, Browman G. Confronting the “gray zones” of technology assessment: evaluating genetic testing services for public insurance coverage in Canada. International Journal of Technology Assessment in Health Care. 2003;2:301–16.

20.	Mykitiuk R. Genetic Services in Ontario: Mapping the Future. Report of the Provincial Advisory Committee on New Predictive Genetic Technologies [Internet]. Advisory Committee on New Predictive Genetic Technologies; 2001. Available from: digitalcommons.osgoode.yorku.ca/reports/101

21.	Burke W, Coughlin SS, Lee NC, Weed DL, Khoury MJ. Application of population screening principles to genetic screening for adult-onset conditions. Genetic Testing. 2001;3:201–11.

22.	Lin JS, Thompson M, Goddard KA, Piper MA, Heneghan C, Whitlock EP. Evaluating genomic tests from bench to bedside: a practical framework. BMC Medical Informatics and Decision Making. 2012;12(117).

23.	Gudgeon JM, McClain MR, Palomaki GE, Williams MS. Rapid ACCE: experience with a rapid and structured approach for evaluating gene-based testing. Genetics in Medicine. 2007;7:473–8.

24.	McClain MR, Palomaki GE, Piper M, Haddow JE. A rapid-ACCE review of CYP2C9 and VKORC1 alleles testing to inform warfarin dosing in adults at elevated risk for thrombotic events to avoid serious bleeding. Genetics in Medicine. 2008;2:89–98.

25.	Pitini E, D’Andrea E, De Vito C, Rosso A, Unim B, Marzuillo C, et al. A proposal of a new evaluation framework towards implementation of genetic tests. Bombard Y, editor. PLoS ONE [Internet]. 2019 Aug 5 [cited 2023 Sep 1];14(8):e0219755. Available from: https://dx.plos.org/10.1371/journal.pone.0219755

26.	Frueh FW, Quinn B. Molecular diagnostics clinical utility strategy: a six-part framework. Expert Review of Molecular Diagnostics. 2014;14:777–86.

27.	Hornberger J, Doberne J, test– CRL developed. Laboratory-Developed Test—SynFRAME: An Approach for Assessing Laboratory-Developed Tests Synthesized from Prior Appraisal Frameworks. Genetic Testing and Molecular Biomarkers. 2012;6:605–14.

28.	UK Genetic Testing Network. UK Genetic Testing Network Testing criteria for molecular genetic tests [Internet]. 2005. Available from: ukgtn.org/gtn/UKGTN-information/dossier.html

29.	Kroese M, Zimmern RL, Farndon P, Stewart F, Whittaker J. How can genetic tests be evaluated for clinical use? Experience of the UK Genetic Testing Network. European Journal of Human Genetics. 2007;9:917–21.
